# Grasshoppers Regulate N:P Stoichiometric Homeostasis by Changing Phosphorus Contents in Their Frass

**DOI:** 10.1371/journal.pone.0103697

**Published:** 2014-08-04

**Authors:** Zijia Zhang, James J. Elser, Arianne J. Cease, Ximei Zhang, Qiang Yu, Xingguo Han, Guangming Zhang

**Affiliations:** 1 State Key Laboratory of Vegetation and Environmental Change, Institute of Botany, Chinese Academy of Sciences, Beijing, China; 2 University of Chinese Academy of Sciences, Beijing, China; 3 School of Life Sciences, Arizona State University, Tempe, Arizona, United States of America; 4 School of Biological Sciences, The University of Sydney, Sydney, Australia; 5 State Key Laboratory of Forest and Soil Ecology, Institute of Applied Ecology, Chinese Academy of Sciences, Shenyang, China; 6 Department of Biology, Graduate Degree Program in Ecology, Colorado State University, Fort Collins, Colorado, United States of America; CNRS, France

## Abstract

Nitrogen (N) and phosphorus (P) are important limiting nutrients for plant production and consumer performance in a variety of ecosystems. As a result, the N:P stoichiometry of herbivores has received increased attention in ecology. However, the mechanisms by which herbivores maintain N:P stoichiometric homeostasis are poorly understood. Here, using a field manipulation experiment we show that the grasshopper *Oedaleus asiaticus* maintains strong N:P stoichiometric homeostasis regardless of whether grasshoppers were reared at low or high density. Grasshoppers maintained homeostasis by increasing P excretion when eating plants with higher P contents. However, while grasshoppers also maintained constant body N contents, we found no changes in N excretion in response to changing plant N content over the range measured. These results suggest that *O. asiaticus* maintains P homeostasis primarily by changing P absorption and excretion rates, but that other mechanisms may be more important for regulating N homeostasis. Our findings improve our understanding of consumer-driven P recycling and may help in understanding the factors affecting plant-herbivore interactions and ecosystem processes in grasslands.

## Introduction

The ecological success and life history of organisms is constrained by a variety of intrinsic and extrinsic factors [1]. For herbivores, diet quality is believed to be one of the most influential extrinsic conditions [2]. Increasingly, nutritional studies have demonstrated that dietary nutrient ratios (*e.g*. N:P) have major effects on growth, reproductive success, and other performance characters in animals [3]. These studies indicate that nutrient balance may be equally or perhaps even more important than the absolute amount of any one nutrient [3], [4]. These findings are central to the theory of ecological stoichiometry, which focuses on the balance of chemical elements and energy in living systems from molecular to global scales [5]–[7], and the related framework, nutritional geometry [4]. While several recent studies have demonstrated that stoichiometric homeostasis may be important in influencing ecological dynamics in various systems [5], [8], [9], and researchers have identified some important pre-ingestive and post-ingestive regulatory mechanisms in insects to achieve their nutritional balances [10], information is still not sufficient on how organisms achieve stoichiometric homeostasis in the face of elementally imbalanced foods. This is especially true for terrestrial consumers under realistic field conditions.

Body N:P ratio has been considered as a central parameter linking key aspects of a species, such as its structural investments, growth rate, and biogeography [11], [12]. Indeed, compared to C:N and C:P ratios, N:P ratios of a species usually have a considerably narrower range of variation [13]–[16]. Importantly, stoichiometric mismatches between consumers and plants due to higher C:nutrient ratios in plants could result in N or P limitation of animal performance, depending on the animal's N:P ratio [17], [18]; thus, regulation of N and P becomes a central issue for herbivorous animals experiencing imbalanced chemical environments [9], [10]. However, N and P differ in their functions and cycling pathways not only within ecosystems but also within organisms [5]. For example, previous work has demonstrated that excess nitrogen eaten by herbivores may be stored within the organisms or excreted as uric acid or in the volatile form of ammonium, whereas P remains in phosphate form [19]–[21]. Furthermore, the relative importance of frass excretion of N and P for regulating N:P stoichiometry in insects remains unclear. We also do not know how the intensity and direction of ecosystem nutrient regulation are affected by grasshopper density, an insight that may be important in understanding when and how insect outbreaks occur.

Grasshoppers are a major component in grassland food webs and are important for controlling plant biodiversity and productivity in grassland ecosystems [2], [22], [23]. Similar to most animals, grasshoppers exhibit greater stoichiometric homeostasis compared to their food plants [24]. Herbivores and their food plants can have interactive effects on each other's C:N:P ratios through various direct and indirect mechanisms [25]. Indeed, our recent work has shown that increasing density in grasshoppers reduces plant standing biomass and dramatically changes the C:N:P stoichiometry of both plants and litterfall [16], possibly due to effects of food plant preference and availability [25]. However, we do not yet know if such density-dependent effects of grasshopper herbivory on plant chemical stoichiometry feedback on grasshopper stoichiometry.

The objective of this study was to answer the following questions: 1) Does N:P stoichiometry in grasshoppers change in response to their own density and plant N:P ratios? If not, then 2) How do grasshoppers achieve this N:P stoichiometric homeostasis? Finally, we assessed the implications of these findings in the context of the overall dynamics of grasshopper and grassland plant interactions.

## Materials and Methods

### Ethics Statement

No specific permissions were required for all these locations, for these lands were not private and the study did not involve endangered or protected species.

### Study site

The field manipulation experiment was carried out in a temperate steppe in the Xilin River Basin in northern China (43°33' N,116°40' E, 1,250 m a.s.l.), which is located at the southeastern part of the Eurasian Steppe. The mean annual temperature in this area is 0.3 °C with mean monthly temperatures ranging from −21.6°C in January to 19.0 °C in July. The mean annual precipitation is 346 mm, more than 80% of which occurs during the growing season from May to September. The soil type is dark chestnut (namely Calcic Luvisols in the FAO classification system) with an average depth of ∼100 cm [16]. Vegetation at the study site is dominated by *Leymus chinensis* and *Stipa grandis* and accompanied by other common species such as *Agropyron cristatum*, *Achnatherum sibiricum*, *Cleistogenes squarrosa*, *Carex korshinskyi*, *Koeleria cristata* and *Allium* sp. Aboveground biomass of these seven species accounts for more than 80% of the total community primary production.

### Study organism

The grasshopper *Oedaleus asiaticus* (formerly *O. decorus asiaticus*), a member of the subfamily Oedipodinae (Orthoptera: Acrididae: Oedipodinae), is a dominant herbivorous insect in the study area, with a population density ranging from <10 individuals m^−2^ before 1990 [26] to >50 individuals m^−2^ after 1990 [27]. It is a mid-sized Acridid species (2.64±0.10 cm in length for males, 3.56±0.24 cm for females). *O. asiaticus* has considerable migratory capacity in its brown-colored phase, making it the dominant locust of north Asian grasslands [28]. Male and female adults of *O. asiaticus* can be visually identified [16].

### Field experiment and sample collecting

During July and August 2007 we established 72 cages within a long-term experimental plot (fenced in 2005 in order to exclude sheep grazing) of the Inner Mongolia Grassland Ecosystem Research Station, Chinese Academy of Sciences. The distance between cages was 1 m. Each cage consisted of a steel-framed and nylon-netted cube (1×1×1 m) with the lower edge sealed against the soil to prevent escape of experimental animals and intrusion of other species. A 0.5×0.5 m zippered window on the upper side of each cage allowed experimental operations such as introduction of grasshoppers, checking of grasshopper densities, and removal of predators (mostly spiders).

We designed a density gradient experiment to simulate grasshopper outbreaks with different intensities. On 20 July 2007, adult *O. asiaticus* were introduced to cages at 6 treatment levels: 0 (control), 2, 4, 8, 16, 32 grasshoppers m^−2^. The sex ratio of each treatment was 1∶1 (male to female, consistent with its natural sex ratio). There were 12 replicates for each treatment, the experiment was based on orthogonal design and had two set of the same number (6×6) of cages. After the experimental grasshoppers were introduced, we checked each cage every three days to confirm the number of living grasshoppers (dead insects were removed and replaced with a similarly-sized animal of the same sex), repair damaged cages, and eliminate spiders and other species of grasshoppers that occurred naturally within the cages. Grasshoppers introduced at the very beginning of grazing were labeled in the pronotum with red paint in order to ensure the sampled insects were those that lived in the cages at a given density condition for 30 days.

On 20 August 2007 (one month after the start), cages were removed. The aboveground standing biomass was harvested by clipping off all plants at the soil surface. According to the methods reliable in our previous work, plants were sorted by species, grasshoppers with red paint markers were carefully removed according to sex and then kept in small cages in lab without feeding for 24 h in order to collect their frass, which was then immediately dried. After that, grasshoppers of both sexes were dried and weighed individually. Each sample of grasshopper used for further chemical analysis was composed of male and female insects with the sex ratio of 1∶1 (male to female). All samples (grasshoppers and plants) were oven dried at 65°C for 48 h and then milled to a fine powder and screened with 0.1-mm mesh before analyzing their N and P contents.

### Chemical Analyses

Total N of plant and insect samples was determined by using a micro-Kjeldahl procedure with sulfuric acid and digestion catalyst. Total phosphorus was analyzed colorimetrically by applying the ammonium molybdate method [29]. All element concentrations in this work were expressed as g kg^−1^ (dry material), and elemental stoichiometry were molar ratios. In the mean time, all analyses are for samples pooled at the plot level.

### Statistical analyses

SPSS version 11.5 (SPSS) was used to analyze our experimental data. T-tests and one-way ANOVAs were applied to compare the N:P stoichiometric difference between different treatments. The relationships between each couple of variables were examined by calculating the Pearson Coefficient, and two-tailed tests of significances were used. Regression analysis was employed to estimate the curves fitted to each couple of experimental variables.

## Results

### Relationships between N and P concentrations in grasshopper body, frass, and food plants

**Figure 1:**
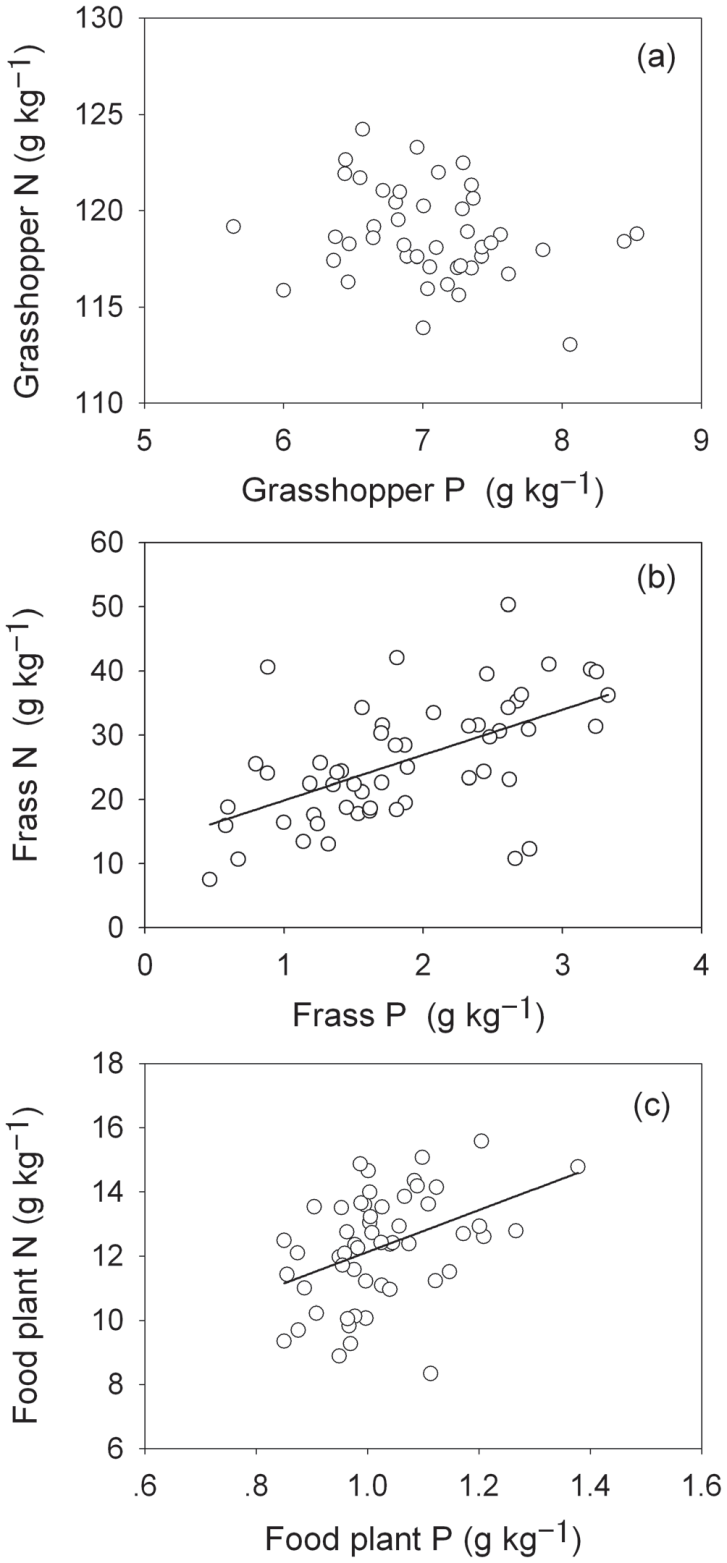
Correlations between N and P concentrations in grasshopper body (a), frass (b), and food plants (c) of grasshopper *Oedaleus asiaticus.*

N and P concentrations in grasshopper bodies were not correlated. In contrast, significant positive linear relationships were found between N and P concentrations in grasshopper frass (*n* = 44, *r* = 0.569, *P*<0.001) and in food plants (*n* = 44, *r* = 0.41, *P* = 0.002) (Fig. 1).

### Contrasting N:P stoichiometric responses in grasshopper body and frass to grasshopper density

**Figure 2:**
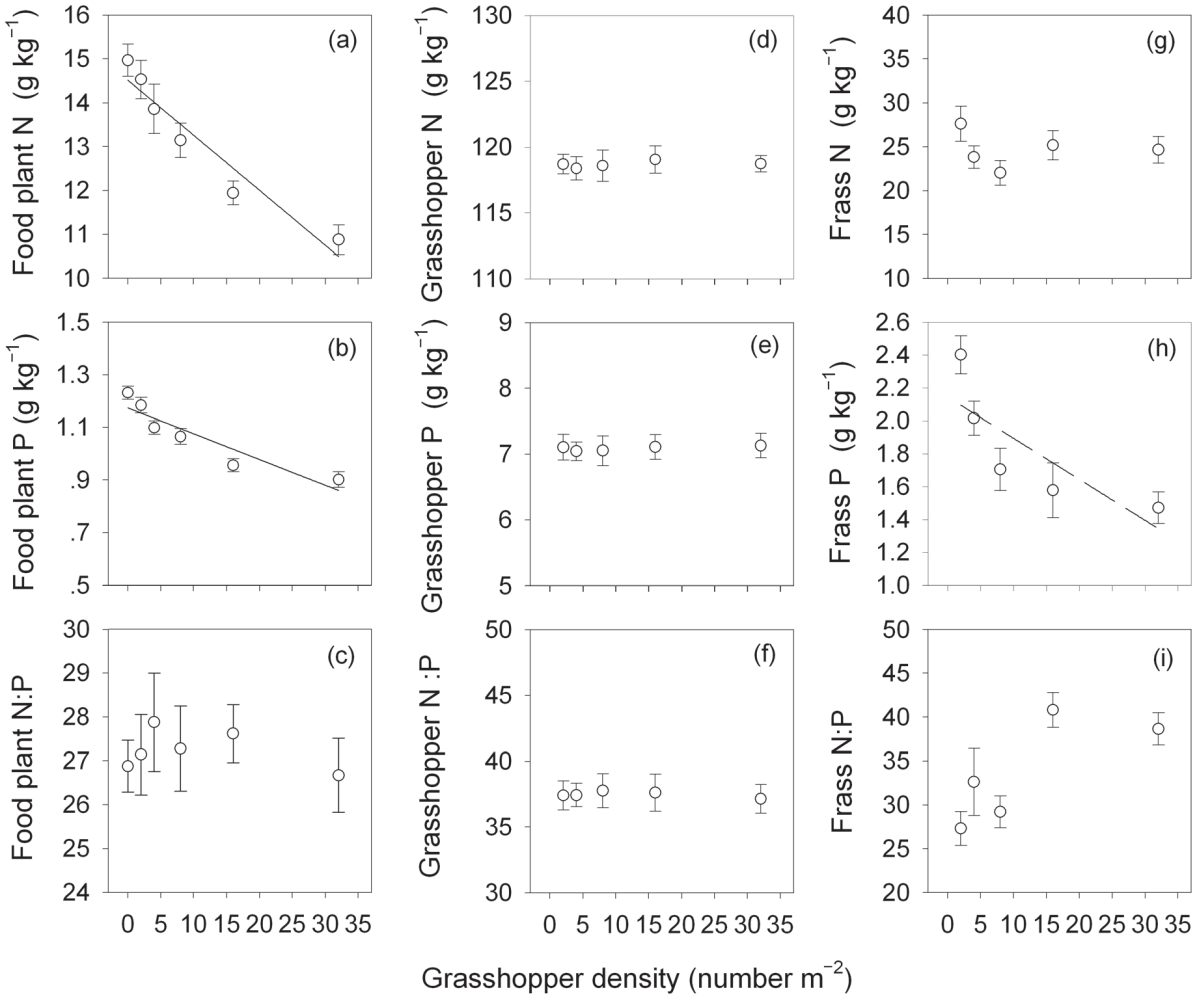
Responses of N and P contents and N:P stoichiometry, of food plants (a–c), grasshopper body (d–f) and frass (g–i), to increasing grasshopper density. Error bars indicate ±1 SE. In (a), a strong negative relationship was found between host plant N concentrations and grasshopper density (ANOVA: *r^2^* = 0.93, *F* = 51.16, *P* = 0.002) and yielded the following equation: y = −0.13 x+14.52. In (b), a strong negative relationship was found between host plant N concentrations and grasshopper density (ANOVA: *r^2^* = 0.86, *F* = 23.59, *P* = 0.008) and yielded the following equation: y = −0.01 x+1.17. In (h), a marginally negative relationship was found between frass P concentrations and grasshopper density (ANOVA: *r^2^* = 0.65, *F* = 5.69, *P* = 0.097) and yielded the following equation: y = −0.025 x+2.14.

Over the density treatments, *O. asiaticus* adults maintained consistent N and P concentrations and N:P ratios, showing strong N:P homeostasis (Fig. 2d–f and Table 1). Along the experimental gradient of grasshopper density, adult *O. asiaticus* N (*n* = 5, *r* = 0.421, *P* = 0.480) and P (*n* = 5, *r* = 0.685, *P* = 0.202) concentrations as well as N:P ratios (*n* = 5, *r* = −0.545, *P* = 0.342) remained similar (Fig. 2d–f). However, frass P concentrations declined as grasshopper density increased, and the correlation between frass P and grasshopper density was marginally significant (*n* = 5, *r* = −0.809, *P* = 0.097) at treatment-level (Fig. 2h) and highly significant (*n* = 45, *r* = −0.467, *P* = 0.001) at quadrat (cage) level (Fig. S1b), and further analysis show that phosphorus decline in frass was occurred when grasshopper density decreased from 2 to 8 individuals per square meter (Fig. S2d–f). In contrast to changes in P, N concentrations of frass did not change appreciably (Fig. 2g and Fig. S1a). Reflecting the changes in N and P contents, frass N:P ratios (*n* = 45, *r* = 0.474, *P* = 0.001) increased with grasshopper densities (Fig. S1c). For food plants, N (*n* = 6, *r* = −0.963, *P* = 0.002) and P (*n* = 6, *r* = −0.925, *P* = 0.008) concentrations both decreased significantly and to similar extents as grasshopper density increased, thus, N:P ratios of food plants (*n* = 6, *r* = −0.358, *P* = 0.485) did not exhibit a strong trend with grasshopper density (Fig. 2a–c).

**Table 1:** Results (*F* and *P* values) of one-way ANOVAs on the effects of grasshopper density on N:P ratio of food plant, grasshopper body and frass, corresponding to Figure 2.

Response variable		Driver: Grasshopper density	
		*F*	*P*
Food plant	N	51.163	0.002
	P	23.589	0.008
	N:P	0.589	0.486
Grasshopper body	N	0.648	0.480
	P	2.648	0.202
	N:P	1.270	0.342
Frass of grasshopper	N	0.033	0.868
	P	5.691	0.097
	N:P	4.166	0.134

### Food plant phosphorus content and N:P stoichiometric variation in grasshopper frass

**Figure 3:**
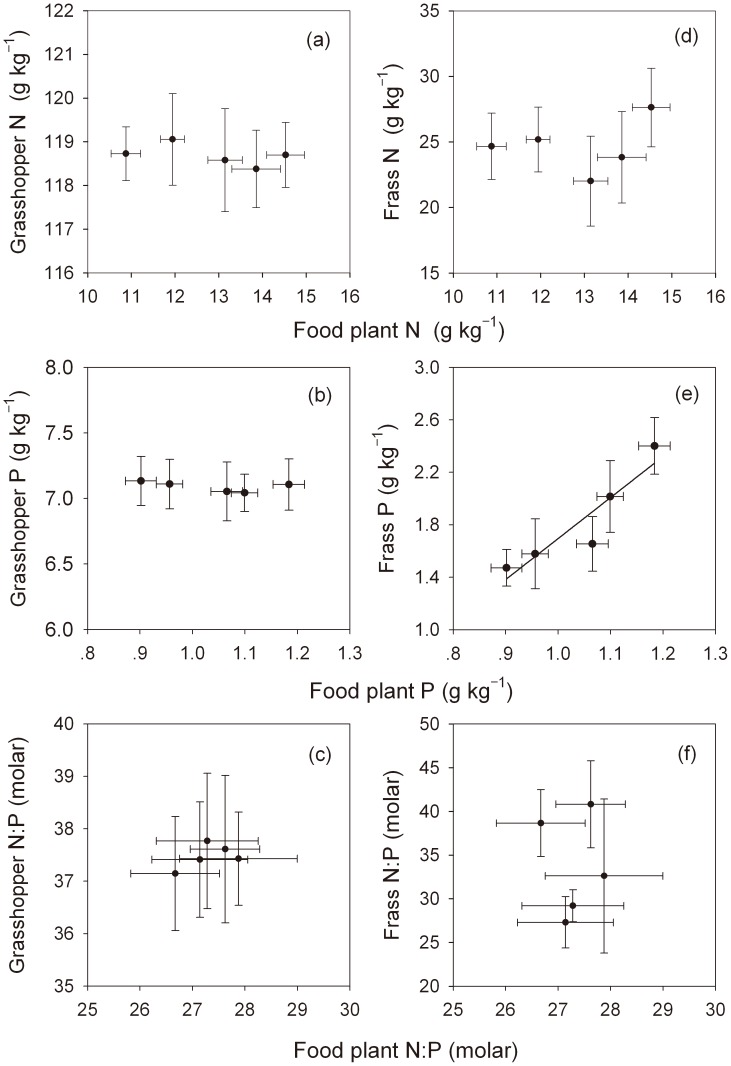
The effects of food plant N:P stoichiometry on N concentrations, P concentrations, N:P ratios in grasshopper body and frass. Error bars indicate ±1 SE. In (e), a strong positive relationship was found between frass and food plant P concentrations (ANOVA: *r^2^* = 0.85, *F* = 17.65, *P* = 0.025) and yielded the following equation: y = 3.13 x−1.43.

After performing the corresponding correlation analyses between the indices of N:P stoichiometry of grasshopper and frass with those of food plants, we found that there were no clear relationships (the correlation was not significant at 0.05 level) for most of these variables with only one exception: frass P concentrations were positively and significantly related to food plant P concentrations (*n* = 5, *r* = 0.944, *P* = 0.016) (Fig. 3e).

### Body size of the grasshopper *O. asiaticus* declined linearly with increasing population density

**Figure 4:**
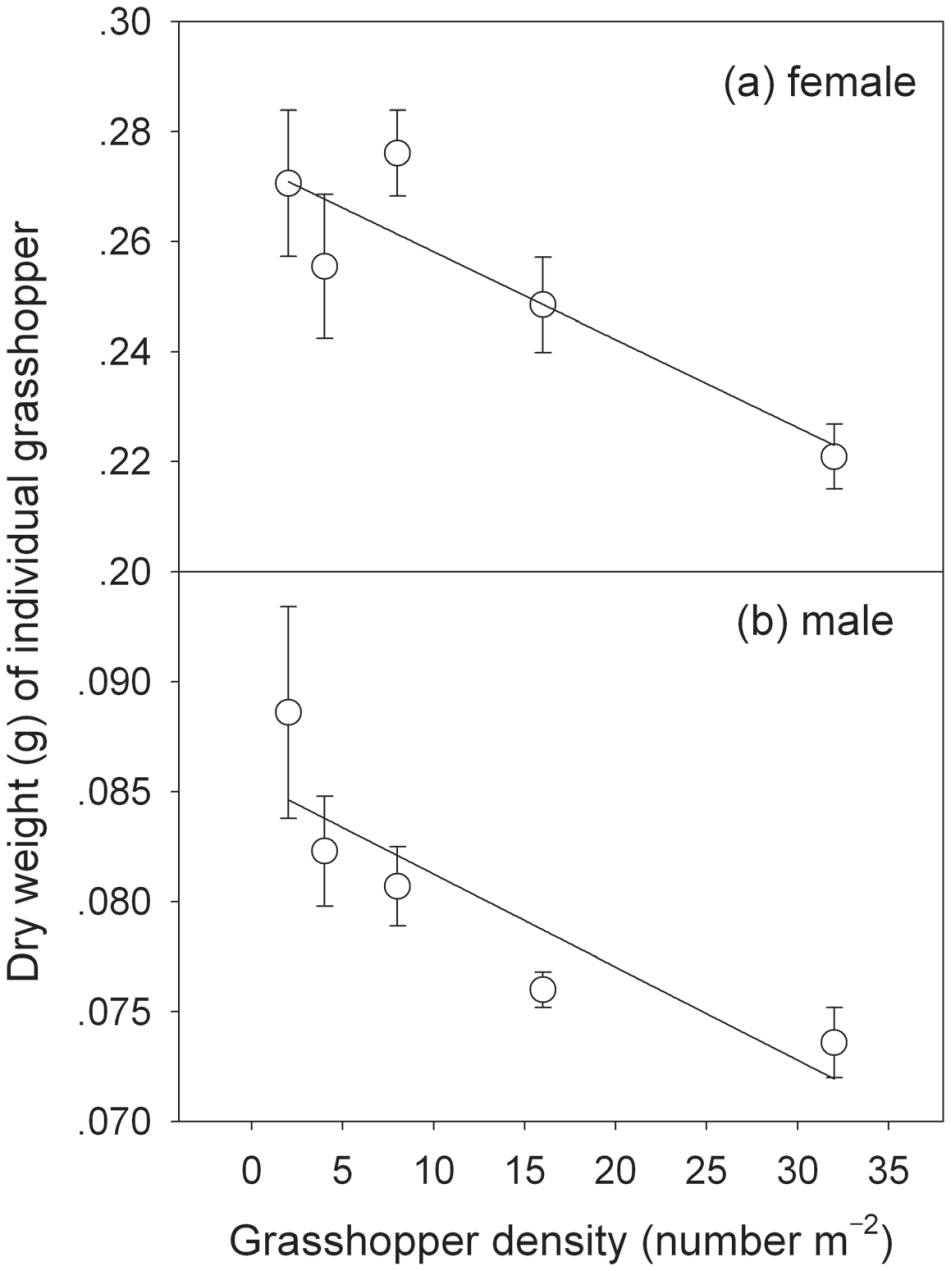
The relationships between female and male body size (expressed by body dry weight) and experimental density in grasshopper *Oedaleus asiaticus*. Error bars indicate ±1 SE. In (a), a negative relationship was found between female body size and grasshopper density (ANOVA: *r^2^* = 0.80, *F* = 12.26, *P* = 0.04) and yielded the following equation: y = −0.0016 x+0.274. In (b), a negative relationship was found between male body size and grasshopper density (ANOVA: *r^2^* = 0.78, *F* = 10.65, *P* = 0.047) and yielded the following equation: y = −0.0004 x+0.085.

In this study, grasshopper body size (expressed as body dry weight) declined significantly in response to increased density (Fig. 4). As grasshopper density increased from 2 to 32 numbers per square meter, dry weight of individual female decreased ∼22% (from 0.271 to 0.221 g) and showed a significant linear relationship (*n* = 5, *r* = 0.896, *P* = 0.039), while dry weight of individual males declined ∼17% (from 0.089 to 0.074 g) and was also negatively related to density (*n* = 5, *r* = 0.883, *P* = 0.047).

## Discussion

### Distinctive responses of consumer versus producer N and P stoichiometry to grasshopper density

The grasshopper *O. asiaticus* in the present study exhibited strong N:P stoichiometric homeostasis under changing food conditions, in this case those generated by experimental manipulations of its own density (Fig. 2d–f). As a grass-feeder, *O. asiaticus* has plentiful food resource in the Inner Mongolian grassland, which is dominated by several species of plants belonging to Family Poaceae [32]. Due to its ecological and evolutionary adaptation to environmental variation, this grasshopper species may be able to obtain optimal nutrient balance to meet its nutritional needs and maintain its body N:P within a narrow range, as seen in other insects [33], [34]. *O. asiaticus* locusts likely adjust their nutrient intake locally by selecting between different plant species and different parts within one individual plant. *O. asiaticus* is a migratory species [28], [30], [31] and thus also has the capacity to balance food intake regionally by large-scale movement. However, our study insects were confined within 1-m^3^ experimental cages (*see* Materials and Methods) and thus had few opportunities to select food plants. This limitation in food selection may have been a contributing factor to decreased body size at high density (Fig. 4).

Contrary to the strong N and P homeostasis in the herbivore, food plant N and P declined with increasing grasshopper density (Fig.2a,b). However, the ratio of N:P in food plants remained unchanged (Fig.2c), indicating again a tightly coupled relationship between N and P in the primary producers in this system, consistent with our previous report [12].

### Consumer P and N regulation

As mentioned above, when grasshopper density increased in the experiment, food plant N and P concentrations both declined substantially (Fig.2a, b). Accordingly, P concentrations in grasshopper frass decreased with decreasing plant P content (and increasing grasshopper density) (Fig.2h and Fig.3e). However, frass N concentrations changed little, even when grasshoppers were eating plants with different N concentrations (Fig.2g and Fig.3d), and the N:P stoichiometry in grasshopper frass was not the functional consequences of that in frass (Fig. S3). These results suggest that *O. asiaticus* maintains P homeostasis primarily by changing P absorption and excretion rates, but that other mechanisms may be more important for regulating N homeostasis.

When confined to no-choice diets, grasshoppers and other insects can post-ingestively regulate dietary N [35]–[37]. Zanotto et al (1993) showed that, when confined to synthetic diets containing different concentrations and balances of protein and carbohydrate, fifth instar *Locusta migratoria* nymphs retained a greater portion of ingested N when on low N diets [36] However, when foraging, herbivores can pre-ingestively regulate protein and carbohydrate balance more strongly than other dietary components, e.g., salts [4], [38]. In our study, *O. asiaticus* may have prioritized regulating N balance pre-ingestively by selecting leaves or leaf parts to meet their macronutrient demands (thus minimizing any change in frass N content) and then post-ingestively regulated P balance. This fits with prior studies on this species that demonstrated changing dietary N and protein content has powerful effects on the growth and viability of *O. asiaticus* [31], suggesting that this locust may have a low capacity to post-ingestively regulate for N imbalance and should prioritize behaviorally regulating N balance when foraging.

### Density-dependent effects on grasshopper body size, and grassland ecosystem stability

Many insects respond to high population density by decreasing body size; possible mechanisms include increased stress, competition for resources, and/or hypoxia [39]. While our study cannot elucidate the mechanism specifically, our data suggest potential for negative feedbacks of high population density to grasshopper performance through decreased dietary P. Our study measured changes in adult body size, so differences based on population density likely reflect differences in structural material and energy stores and/or egg and sperm production. Here in the present work we did not expand our investigation to these physiological variations in grasshoppers, although some ecosystem level implications may be disentangled.

Increasing grasshopper density has been demonstrated to exert considerable pressure on plant growth and ecosystem productivity [16], [40]. Under a situation of grasshopper population outbreaks, such as the high density treatment in this experiment, decreasing P concentrations and thus increasing N:P ratios in frass (Fig.2i) may lead to more lower availability of soil P relative to N (skewed consumer-driven nutrient recycling). This high N:P frass may stimulate plant growth, which is generally limited by N availability in Inner Mongolia grassland [41]. However, it is important to note that our findings are based on a single field season and involved only adult locusts, limiting our ability to generalize to other conditions. Nevertheless, given that grasshopper and locust plagues can occur in even shorter time scales at given locality [42]–[44] and may result from a swarm of adults settling in a new area (as was simulated by our study), our findings provide useful insights into grasshopper-plant-soil interactions. In addition, global disruption of biogeochemical cycles due to various human activities may induce significant alterations in N and P cycling and their coupling relationships in terrestrial ecosystems [45]–[47], potentially disrupting grassland plant-herbivores relationships. Thus, a more complete understanding of the stoichiometric impacts of grasshopper-plant interactions on grassland biogeochemical cycling is needed and may be informed by our study.

## Conclusions and Implications

Our experiment sheds some light on how at least one grasshopper species, *O. asiaticus*, maintains stoichiometric homeostasis in the face of changes in food abundance and elemental composition, highlighting changes in rates of absorption and excretion as an important physiological mechanism in maintaining the stability of body P content. While the ecological significance of frass N production has been reported by several authors [45], we still know little about the effects of P release to soil via frass. To our knowledge, this study is the first to investigate the role of excretion in regulating the P homeostasis in an Acridid herbivore under ecologically realistic conditions. The decreasing amount of frass P input to soil nutrient pools that we document may have significant implications for plant-herbivore dynamics and ecosystem functioning.

## Supporting Information

Figure S1 Effects of grasshopper density on frass N:P stoichiometry. In (b), a negative relationship was found between frass P concentration and grasshopper density (ANOVA: *r^2^* = 0.218, *F* = 11.98, *P* = 0.001) and yielded the following equation: y = −0.029 x+2.23. In (c), a positive relationship was found between frass N:P ratio and grasshopper density (ANOVA: *r^2^* = 0.225, *F* = 12.48, *P* = 0.001) and yielded the following equation: y = 0.48 x+25.87.
(DOC)

Figure S2 Comparision of the effects of different grasshopper densities on N:P ratio of food plant, grasshopper body and frass. Error bars indicate ±1 SE. Different letters above different bars mean significant difference at 0.05 level, based on one-way ANOVAs.
(DOC)

Figure S3 Relationship of N:P stoichiometry between grasshoppers body and frass. Error bars indicate ±1 SE. In (a) for N analysis, *r* = 0.378, *P* = 0.530; In (b) for P analysis, *r* = −0.258, *P* = 0.675; In (c) for N:P analysis, *r* = −0.279, *P* = 0.650.
(DOC)
