# Supplementary material for: Grasshoppers Regulate N:P Stoichiometric Homeostasis by Changing Phosphorus Contents in Their Frass
Source: PLoS One. 2014 Aug 4;9(8):e103697. doi: 10.1371/journal.pone.0103697 (PMC4121213; doi:10.1371/journal.pone.0103697)
Supplement: Figure S1 — Effects of grasshopper density on frass N:P stoichiometry. In (b), a negative relationship was found between frass P concentration and grasshopper density (ANOVA: r2 = 0.218, F = 11.98, P = 0.001) and yielded the following equation: y = −0.029 x+2.23. In (c), a positive relationship was found between frass N:P ratio and grasshopper density (ANOVA: r2 = 0.225, F = 12.48, P = 0.001) and yielded the following equation: y = 0.48 x+25.87. (DOC) [file pone.0103697.s001.doc]

**Supporting Information：**Figure S1

**For** Zhang *et. al.* Grasshoppers regulate N:P stoichiometric homeostasis by changing phosphorus content in their frass

Figure S1. Effects of grasshopper density on frass N:P stoichiometry. In (b), a negative relationship was found between frass P concentration and grasshopper density (ANOVA: *r^2^* = 0.218, *F* = 11.98, *P* = 0.001) and yielded the following equation: y = – 0.029 x + 2.23. In (c), a positive relationship was found between frass N:P ratio and grasshopper density (ANOVA: *r^2^* = 0.225, *F* = 12.48, *P* = 0.001) and yielded the following equation: y = 0.48 x + 25.87.
