# Supplementary material for: Grasshoppers Regulate N:P Stoichiometric Homeostasis by Changing Phosphorus Contents in Their Frass
Source: PLoS One. 2014 Aug 4;9(8):e103697. doi: 10.1371/journal.pone.0103697 (PMC4121213; doi:10.1371/journal.pone.0103697)
Supplement: Figure S2 — Comparision of the effects of different grasshopper densities on N:P ratio of food plant, grasshopper body and frass. Error bars indicate ±1 SE. Different letters above different bars mean significant difference at 0.05 level, based on one-way ANOVAs. (DOC) [file pone.0103697.s002.doc]

**Supporting Information:** Figure S2

**For** Zhang *et. al.* Grasshoppers regulate N:P stoichiometric homeostasis by changing phosphorus content in their frass

Figure S2. Comparision of the effects of different grasshopper densities on N:P ratio of food plant, grasshopper body and frass. Error bars indicate ± 1 SE. Different letters above different bars mean significant difference at 0.05 level, based on one-way ANOVAs.
