# Supplementary material for: Grasshoppers Regulate N:P Stoichiometric Homeostasis by Changing Phosphorus Contents in Their Frass
Source: PLoS One. 2014 Aug 4;9(8):e103697. doi: 10.1371/journal.pone.0103697 (PMC4121213; doi:10.1371/journal.pone.0103697)
Supplement: Figure S3 — Relationship of N:P stoichiometry between grasshoppers body and frass. Error bars indicate ±1 SE. In (a) for N analysis, r = 0.378, P = 0.530; In (b) for P analysis, r = −0.258, P = 0.675; In (c) for N:P analysis, r = −0.279, P = 0.650. (DOC) [file pone.0103697.s003.doc]

**Supporting Information 3：**Figure S3

**For** Zhang *et. al.* Grasshoppers regulate N:P stoichiometric homeostasis by changing phosphorus content in their frass

Figure S3. Relationship of N:P stoichiometry between grasshoppers body and frass. Error bars indicate ± 1 SE. In (a) for N analysis, *r* = 0.378, *P* = 0.530; In (b) for P analysis, *r* = - 0.258, *P* = 0.675; In (c) for N:P analysis, *r* = - 0.279, *P* = 0.650.
